# Complete Genome of Geobacter pickeringii G13^T^, a Metal-Reducing Isolate from Sedimentary Kaolin Deposits

**DOI:** 10.1128/genomeA.00038-15

**Published:** 2015-03-05

**Authors:** Jonathan P. Badalamenti, Daniel R. Bond

**Affiliations:** Department of Microbiology and BioTechnology Institute, University of Minnesota, Saint Paul, Minnesota, USA

## Abstract

We used PacBio sequencing to assemble the genome of the pristine freshwater isolate Geobacter pickeringii G13^T^ into a single 3,618,700-bp circular chromosome polished to 99.999% accuracy (quality value [QV], 50). This isolate shares several features with other Geobacter spp., including genes for degradation of aromatics and an abundance of multiheme *c*-type cytochromes.

## GENOME ANNOUNCEMENT

Geobacter spp. play important roles in coupling organic matter degradation to metal reduction in sediment and subsurface environments. Genome sequencing efforts have primarily focused on isolates from aquatic sediments, subsurface bioremediation sites, and electricity-producing bioreactors (1–4). Geobacter pickeringii, a freshwater isolate recovered from a pristine, uncontaminated Fe(III)-rich kaolin deposit, is of interest for its wide repertoire of reported electron donors and acceptors used for anaerobic respiration, including the rare ability to utilize methanol (5).

Closed assemblies are available for 7 Geobacter isolates in GenBank, and these provide important structural information typically lacking in fragmented assemblies. For single-contig assembly of G. pickeringii, we leveraged Pacific Biosciences long-read sequencing to span repetitive elements without relying on reads from complementary platforms (6). Approximately 10 µg of genomic DNA from enzymatically lysed, stationary-phase G. pickeringii cells was prepared for continuous long reads using a 20-kb insert target and electrophoretically size selected with a 4-kb cutoff using Blue Pippin (Sage Science). Reads were captured as 120-min movies from 5 SMRT cells (P4-C2 chemistry) on a PacBio RS II sequencer (Mayo Clinic Bioinformatics Core, Rochester, MN).

Raw reads (~1 Gbp total) were filtered to remove SMRT bell adapters and short (<100 bp) and low quality reads (<80% accuracy) using SMRT Analysis v. 2.1 within the Amazon EC2 Elastic Compute Cloud. *De novo* assembly was performed with ~30× coverage of self-corrected long reads with a length cutoff of 10,183 bp as determined automatically with HGAP (7). Contig lengths from the first assembly were summed to estimate genome size and rerun HGAP, producing a single contig with self-overlapping ends. Overlaps were manually trimmed and the circularized contig was subjected to two successive rounds of polishing with Quiver using the entire read set (250× coverage) to remove remaining indels (7) and produce a single, 3,618,700-bp circular chromosome with uniform coverage, G+C content of 62.27%, and consensus concordance of 99.999% (quality value [QV], 50). The chromosome was manually reoriented to begin at the replication origin as predicted using Ori-Finder (8) before annotation via the NCBI Prokaryotic Annotation Pipeline.

Despite being isolated from an uncontaminated environment, G. pickeringii contains a full suite of genes for anaerobic oxidation of aromatic compounds and a putative *hgcAB* gene cluster (GPICK_04815—GPICK_04820) responsible for mercury methylation (9). G. pickeringii also contains a complete tricarboxylic acid (TCA) cycle, 60 predicted histidine kinases, and 56 multiheme *c*-type cytochromes, similar to Geobacter sulfurreducens (72) and Geobacter metallireducens (60). Long-read assembly successfully resolved 3 rRNA gene operons and a near perfect duplication of a 7.4-kb heterodisulfide reductase gene cluster. In support of the G. pickeringii methanol utilization phenotype (5), genes linked to methanol oxidation in Rhodococcus erythropolis N9T-4 (10) occurred as a cluster in G. pickeringii (GPICK_06655—GPICK_06695) upstream of a biosynthetic pathway predicted to synthesize an electron transfer cofactor (mycofactocin) used in methanol oxidation (11).

### Nucleotide sequence accession numbers.

The assembly and annotation have been deposited in GenBank under accession number CP009788. Raw PacBio reads and base modification data have been deposited to the NCBI Sequence Read Archive under accession number SRX796422.
